# Precision medicine based on surgical oncology in the era of genome‐scale analysis and genome editing technology

**DOI:** 10.1002/ags3.12059

**Published:** 2018-01-24

**Authors:** Shinji Tanaka

**Affiliations:** ^1^ Department of Molecular Oncology Tokyo Medical and Dental University Tokyo Japan

**Keywords:** cancer stemness, genome editing, mitotic instability, molecular subtype, precision medicine

## Abstract

Accumulated evidence suggests that multiple molecular and cellular interactions promote cancer evolution in vivo. Surgical oncology is of growing significance to a comprehensive understanding of the malignant diseases for therapeutic application. We have analyzed more than 1000 clinical samples from surgically resected tissue to identify molecular biomarkers and therapeutic targets for advanced malignancies. Cancer stemness and mitotic instability were then determined as the essential predictors of aggressive phenotype with poor prognosis. Recently, whole genome/exome sequencing showed a mutational landscape underlying phenotype heterogeneity in caners. In addition, integrated genomic, epigenomic, transcriptomic, metabolic, proteomic and phenomic analyses elucidated several molecular subtypes that cluster in liver, pancreatic, biliary, esophageal and gastroenterological cancers. Identification of each molecular subtype is expected to realize the precise medicine targeting subtype‐specific molecules; however, there are obstacle limitations to determine matching druggable targets or synthetic lethal interactions. Current breakthroughs in genome editing technology can provide us with unprecedented opportunity to recapitulate subtype‐specific pathophysiology in vitro and in vivo. Given a great potential, on‐demand editing system can design actionable strategy and revolutionize precision cancer medicine based on surgical oncology.

## 1 INTRODUCTION

**Figure 1:**
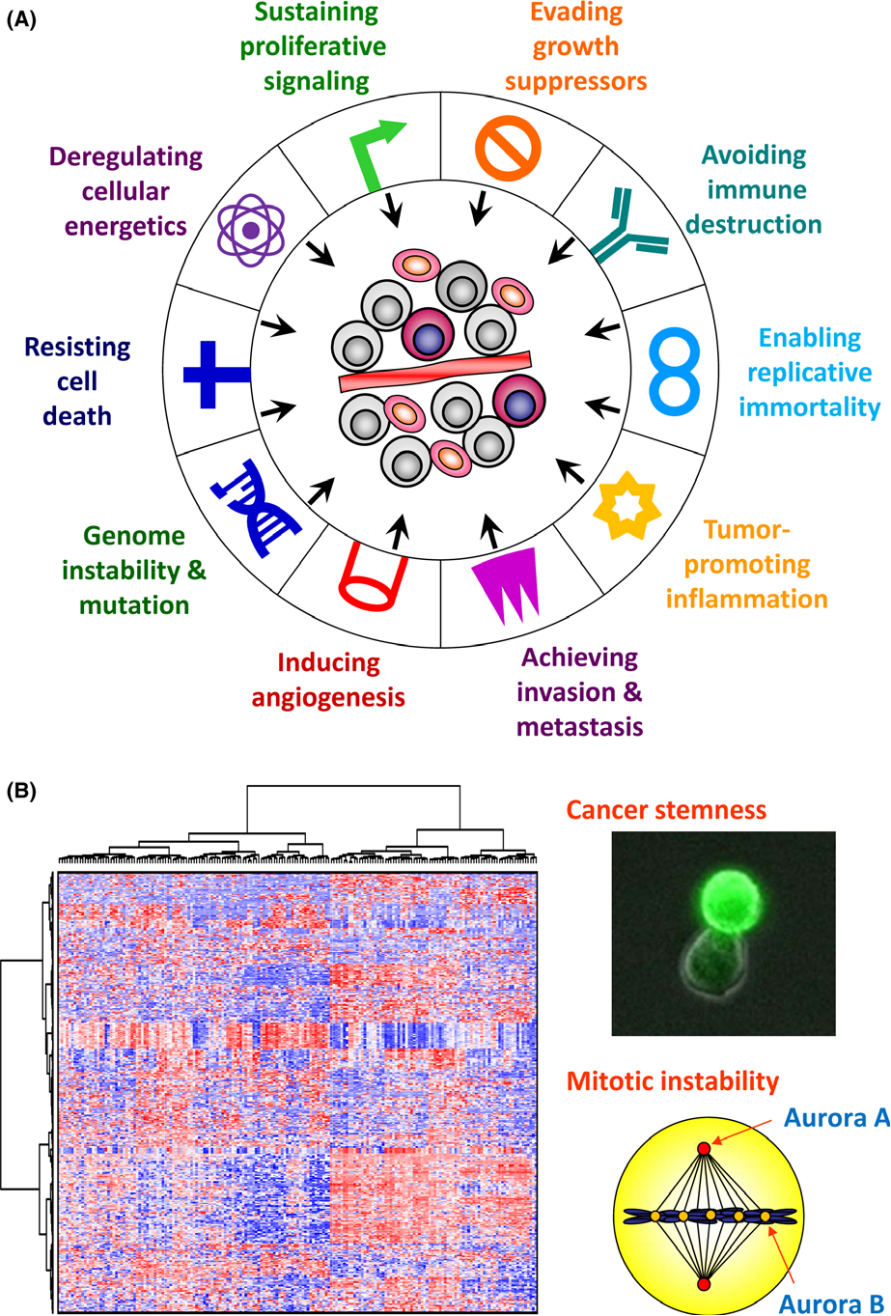
Hallmarks and transcriptomics of cancer. A, The hallmark catalog of cancer phenotypes is a manifestation of 10 essential alterations in cell physiology that collectively dictate malignant growth.^3^ B, Genome‐scale transcriptomic analysis with microarray identified the stemness pathway and mitotic abnormality as the main regulators of hepatocellular carcinoma with poor prognosis

Heterogeneity is one of the essential characteristics of malignancies.^1^ Regarding intertumor heterogeneity, striking variability exists in biological characteristics including proliferation rate, cell‐cell interaction, metastatic tendency and even response to cancer treatment.^2^ Various hallmarks of cancer phenotype are proposed to collectively promote survival and progress in vivo.^3^ They consist of sustaining proliferative signaling, evading growth suppressors, enabling replicative immortality, resisting cell death, deregulating cellular energetics, genome instability and mutation within cancer cells, as well as avoiding immune destruction, tumor‐promoting inflammation, inducing angiogenesis, achieving invasion and metastasis in the tumor‐host interactions (Figure 1). Not only cancer cells themselves, but also complex intercommunications in the tumor microenvironments should contribute to in vivo evolution of malignant diseases, indicating essential and irreplaceable roles of clinical tissue samples resected surgically.^4^, ^5^ Recent advances of subtype stratification have been achieved by integrative studies of transcriptomics with genomics, epigenomics, metabolomics proteomics and phenomics using surgical specimens and clinicopathological data.^6^ In this review, the current strategies for unparalleled challenge of subtype‐guided treatment that links molecular properties to targeted therapy, and perspectives of the future of precision cancer medicine with genome editing technology, are discussed. As these potentials could be augmented by gastroenterological surgery, the concept of precision medicine based on surgical oncology is of importance in cancer treatment.

## 2 CANCER STEMNESS REPROGRAMMING AS THERAPEUTIC RESISTANCE

**Figure 2:**
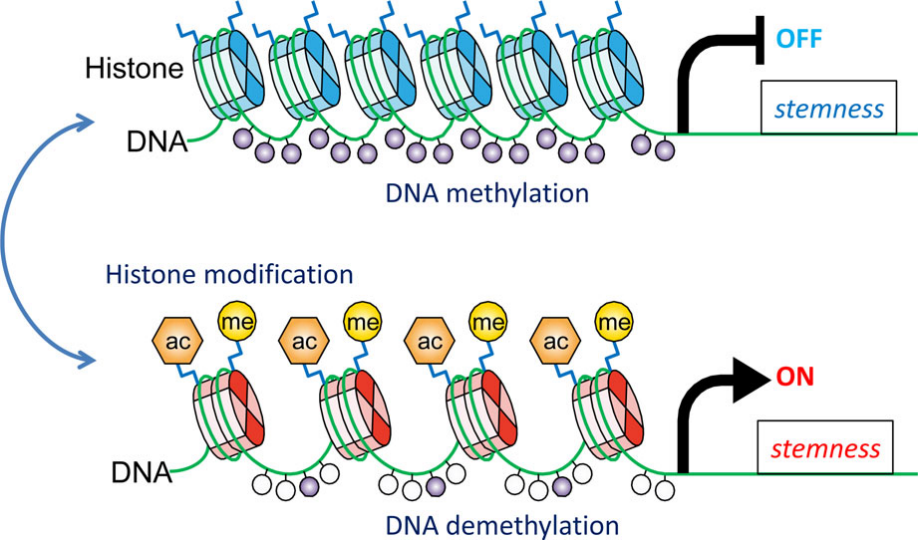
Histone modification to switch the cancer stem cell (CSC) phenotype on and off. Stemness gene promoter region is enriched with active histone marks H3K4me3 and H3K27ac showing open chromatin states that are frequently coexistent with CpG demethylation in stemness‐phenotype cells

A variety of phenotypic hallmarks of cancer is characterized as tumor heterogeneity in vitro and in vivo.^3^ These heterogeneity patterns can be determined by molecular analyses, and transcriptomics using bulk tumor tissues are suitable for clustering to better understand the transcriptional networks that underpin the tumor microenvironment.^7^, ^8^ First, we carried out genome‐wide transcriptome analysis on surgically resected samples using a microarray technique (Figure 2). Subsequently, the stemness pathway^9^ and mitotic abnormality^10^ were identified as the main regulators of hepatocellular carcinoma (HCC) with poor prognosis.

The stemness phenotype comprises the essential component of intractable cancers.^11^ Cancer cells with stem‐like properties, called cancer stem cells (CSC), feature the ability of self‐renewal and pluripotency to hierarchically organize tumor initiation and maintenance.^12^ CSC lying at the apex of the hierarchy are intrinsically resistant to chemotherapeutic agents, and function as a source to metastasizing and relapsing. “Self‐renewal” is theoretically based on asymmetric divisions of stem cells that give rise to one cell of the stem cell potency and another stimulated to differentiate further into non‐stem cell types.^13^ In our recent studies, the proteasome‐independent character of the stem cell fate (degron) was used for fluorescent visualization of CSC subpopulations in human HCC^14^ as well as in pancreatic cancer^13^ and colorectal cancer cells.^15^ Noteworthy, this system to distinguish CSC from non‐CSC showed asymmetric cell division, “self‐renewal” sphere formation in a real‐time manner, and over 1000‐fold increase in tumorigenicity with heterogenic expansion in vivo.^14^, ^15^, ^16^ As CSC might play a fundamental role in these awful malignant behaviors, investigations of the molecular targets of CSC may show particularly effective therapeutic approaches.^12^ We showed EpCAM stemness marker as one of the therapeutic targets of human HCC in vitro and in vivo.^17^ As stem cell features are addictive to p53 inactivation,^18^ CSC‐targeted therapy might be more effective on *TP53*‐mutated subtype of HCC.^19^

Chromatin dynamics play an essential role in stem cell fate determination.^20^ We showed that metastatic potentials and gene expression profiles of CSC are regulated by histone modifications for open‐bivalent‐closed chromatin statuses.^21^ In our recent studies, sorafenib‐resistant HCC was shown to acquire in vivo CSC features with histone modification.^22^ We identified that H3K4me3 and H3K27ac levels were globally elevated in HCC cells surviving under the inhibition of angiogenesis, providing the first evidence that dynamic epigenetic states of CSC could be influenced by modulating the tumor microenvironment in vivo. Cumulative findings indicating that an open chromatin state contributes to maintenance of pluripotency in stem cells^23^ are thereby consistent with our observation of similar epigenetic alterations during acquisition of stemness and drug resistance. Such diversity makes the investigation and treatment of cancers complicated. CSC are believed to be responsible for resistance to conventional therapies and metastatic abilities in clinical practice.^12^ Epigenetic addictions of CSC might be promising properties for development of advanced cancer therapy. In our ongoing research, single‐cell and ATAC‐seq analyses are applied to identify the master molecule of epigenetic reprogramming as a therapeutic target for the CSC phenotype of resistant HCC.

## 3 MITOTIC INSTABILITY AND PUNCTUATED EQUILIBRIUM

One of the major difficulties in the treatment of HCC is the high frequency of tumor recurrence even after curative resection. According to our clinical studies, not the recurrence itself, but the rapid and lethal recurrence pattern has critical effects on prognosis of the patient with HCC.^24^ In this regard, Aurora mitotic abnormality was shown as the essential pathway for such an aggressive phenotype of HCC.^7^

Aurora kinases are serine/threonine kinases that play major roles in chromosomal alignment and segregation during mitosis and cytokinesis.^25^ Aurora A localizes to the spindle poles, whereas Aurora B localizes to the midbody of the central spindle during mitosis. Aurora A regulates centrosome maturation and separation and bipolar spindle assembly. Aurora A phosphorylates and activates polo‐like kinase 1 (PLK1), promoting cyclin‐dependent kinase activation and mitotic entry. Aurora B controls chromosome bi‐orientation as a member of the chromosome passenger complex as well as proper execution of cytokinesis.

Aurora kinases A and B interact with and phosphorylate p53 at distinct residues, and regulate p53 transactivation activity as well as stability through the ubiquitination‐mediated proteasome pathway, resulting in abrogation of the DNA damage checkpoint and induction of cell death responses.^26^, ^27^ According to our studies, treatment with Aurora B inhibitor in vitro and in vivo resulted in polyploidy and cell death by mitotic catastrophe.^28^ In addition, sequential combination treatment with Aurora B inhibitor (barasertib) followed by Bcl2/xL inhibitor (navitoclax) significantly suppressed orthotopic liver tumors.^29^ In the recent studies, Dauch et al^30^ reported that *TP53*‐mutated human HCC cells were specifically sensitive to Aurora A inhibitor, thus suggesting a novel therapeutic strategy for this subtype of human HCC. These preclinical studies indicate that Aurora is a promising molecular target “Achilles' heel” for the treatment of aggressive HCC.^31^

What is the critical role of the Aurora mitotic pathway in cancer progression? Our microarray‐based comparative genomic hybridization (array‐CGH) analysis on clinical samples showed that genomic instability was closely related to Aurora B overexpression in HCC.^10^ The fraction of genome altered (FGA) in Aurora B‐positive cases was significantly higher than that in Aurora B‐negative cases (*P* = .009), suggesting that overexpression of Aurora B may contribute to genomic instability in HCC. Indeed, in vitro overexpression of Aurora A caused inactivation of the spindle assembly checkpoint during mitosis, leading to polyploidy and centrosome amplification.^32^ Similarly, overexpression of Aurora B caused defective chromosome separation during mitosis, leading to aneuploidy with mitotic errors.^33^ Amon's group and our collaborators clarified that poly‐or aneuploidy is potentially critical for the fate of malignant evolution.^34^, ^35^

**Figure 3:**
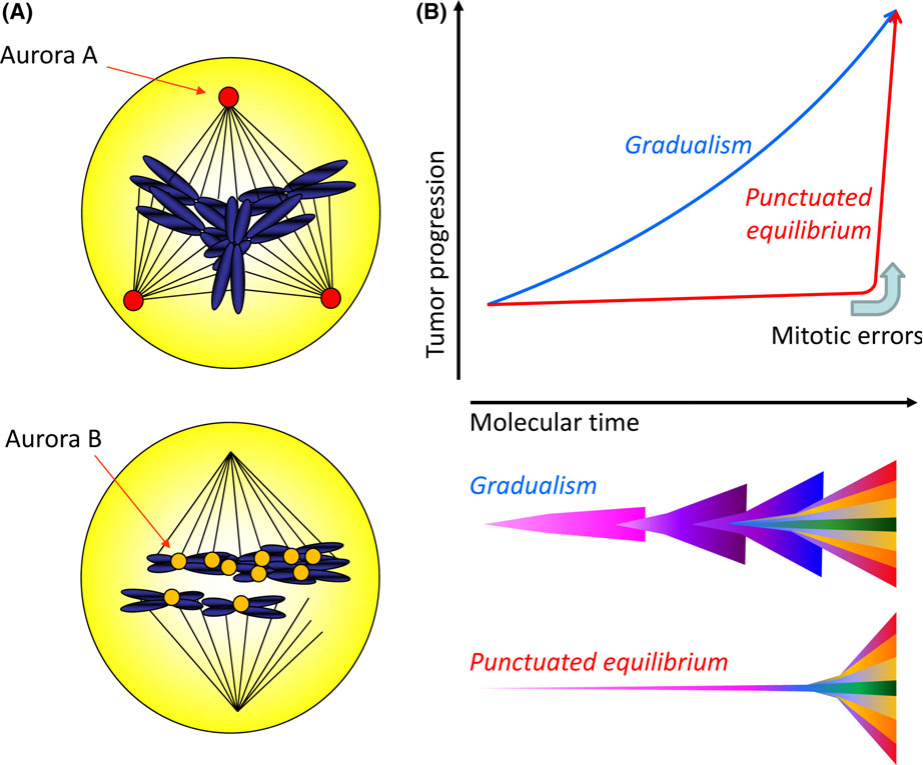
Mitotic instability and punctuated equilibrium. A, Aberrant expression of Aurora A and B induced poly‐ or aneuploidy with mitotic errors.^32^, ^33^ B, Evolution models for classical gradualism (blue) and alternatively punctuated equilibrium (red).^37^ In the gradualism model, multiple transforming events are independently required for tumor development. In the punctuated equilibrium model, tumor development can be divided into two major events: the cancer‐initiating event and then the revolutionary‐chromothripsis event is triggered catastrophically by poly‐ or aneuploidy with mitotic errors^36^

Chromosome segregation errors can lead to DNA damage and chromosomal aberrations such as poly‐or aneuploidy which is linked to chromothripsis, a new class of complex catastrophic chromosomal rearrangement.^36^ Chromothripsis is a dramatic event that results in the pulverization of one or a few select chromosomes followed by their highly error‐prone re‐stitching. This leads to extensive chromosome rearrangements, which often include deletions, non‐balanced translocations, duplications, and inversions. Recently, chromothripsis associated with mitotic errors was identified as the principal evolutionary trajectory in aggressive cancer progression such as pancreatic adenocarcinoma.^37^ The consequence of mitotic errors is not sequential but simultaneous, indicating “punctuated equilibrium”, rather than “gradualism” in a subset of cases (Figure 3B). The innovative investigations of malignant evolution will be essential to guide therapeutic strategies for lethal cancers.

## 4 MOLECULAR SUBTYPES AND GENOME EDITING TECHNOLOGY

**Figure 4:**
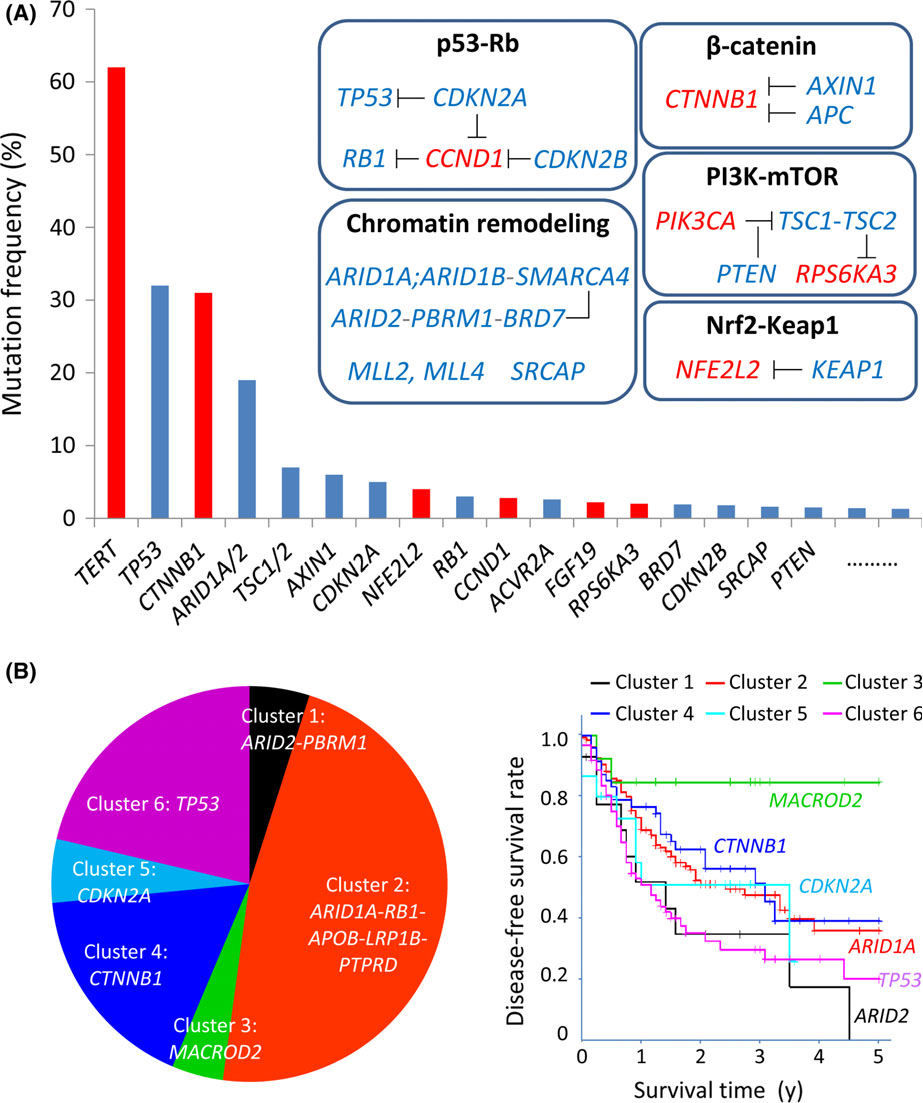
Landscape of altered genes and clusters in hepatocellular carcinoma (HCC). A, Bar plot and main pathways indicating the major events for oncogenes (red) and tumor suppressor genes (blue) altered frequently in HCC.^43^, ^44^ B, Six mutation clusters and Kaplan‐Meier plot of disease‐free survival of HCC patients^46^

The genomic mutational landscape might contribute to practical comprehension of tumor heterogeneity.^38^ Decades ago, manual DNA sequencing detected individual mutations in *TP53* (30%~) and *CTNNB1* encoding beta‐catenin (~30%) in human HCC.^39^ We have previously reported the closed relationship between *TP53* mutations and HCC progression,^40^ and the carcinogenic significance of Wnt/beta‐catenin signaling pathways.^41^, ^42^ In recent years, next‐generation sequencing for whole exome analysis elucidated that more than 60% of HCC carries aberrant activation of *TERT* (telomere reverse transcriptase) through promoter mutations, viral integrations or focal amplifications (Figure 4A).^43^ SWI/SNF chromatin‐remodeling complex was identified as another candidate of the major driver mutations in HCC.^10^ Approximately 20%‐30% of HCC carries genomic aberrations encoding SWI/SNF subunits such as ARID1A, ARID2, and PBRM1.^43^, ^44^ The SWI/SNF enzymatic complex functions as an ATP‐dependent helicase to disrupt histone‐DNA contacts to create a loop of DNA as the essential step required for DNA replication and transcription as well as DNA repair.^45^

It is interesting that mutually exclusive patterns of gene mutations are recognized between *TP53* and *CTNNB1*, or between *ARID1A* and *ARID2*.^43^ Whole genome analysis by Fujimoto et al^46^ identified the HCC mutational landscape that can be subclassified into six mutational clusters: cluster 1: *ARID2‐PBRM1* (4.9%); cluster 2: *ARID1A‐RB1‐APOB‐LRP1B‐PTPRD* (47.2%); cluster 3: *MACROD2* (4.2%); cluster 4: *CTNNB1* (17.1%); cluster 5: *CDKN2A* (5.2%); and cluster 6: *TP53* (21.3%). In particular, cases in the *ARID2*‐mutated cluster showed the poorest prognosis after curative operation (Figure 4B).

Characterization of genomic mutations is required for clarification of the molecular significance but remains challenging as a result of complex manipulation. To overcome this limitation, novel genome‐editing technologies have been developed to manipulate the genome precisely by deletion, insertion, or modification of targeted loci specifically. Breakthroughs in clustered regularly interspaced short palindromic repeats (CRISPR)‐mediated genome editing technology provide us with unparalleled opportunity to bring precision medicine to on‐demand modification.^47^ CRISPR/Cas9 technology has progressed swiftly, allowing its common use to investigate genetic function in preclinical studies.

**Figure 5:**
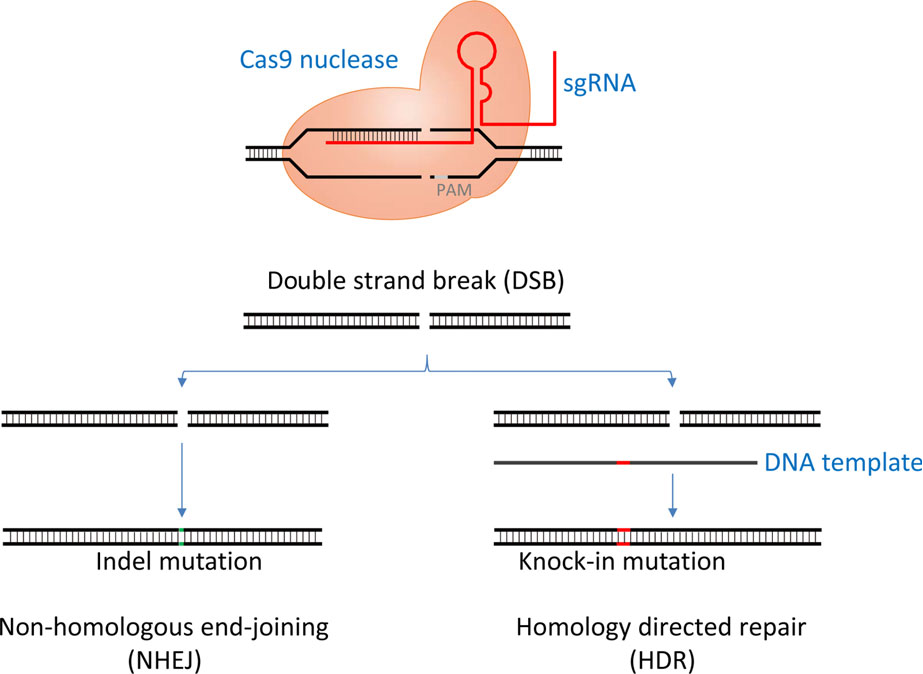
Clustered regularly interspaced short palindromic repeats (CRISPR)/Cas9 genome‐editing system.^48^ Cas9 nuclease protein precisely cleaves the target DNA by use of short single‐guide RNA (sgRNA), immediately followed by species‐dependent protospacer adjacent motif (PAM). After generation of a double‐strand break (DSB), non‐homologous end joining (NHEJ) repair pathway induces indel mutation, resulting in gene knock‐out. Additionally, a homologous DNA template enables homology‐directed repair (HDR) pathway, resulting in gene knock‐in

The essential mechanism of the CRISPR/Cas9 system consists simply of two or three essential components (Figure 5).^48^ Cas9 protein recognizes the DNA‐binding site through RNA‐DNA interactions mediated by short single‐guide RNA (sgRNA), which can be easily designed. The nuclease domain of Cas9 cleaves both strands of the target DNA at −3 nucleotides before protospacer adjacent motif (PAM) sequence, generating a double‐strand break (DSB). Then, non‐homologous end joining (NHEJ) repair pathway can result in the introduction of insertion‐deletion (indel) mutations that can lead to a frameshift, the introduction of a premature stop codon and, consequently, gene knock‐out (Figure 5). Alternatively, in addition to Cas9 and sgRNA, a homologous DNA template enables a homology‐directed repair (HDR) pathway that can introduce precise genetic modifications (e.g. knock‐in mutations).^49^

**Figure 6:**
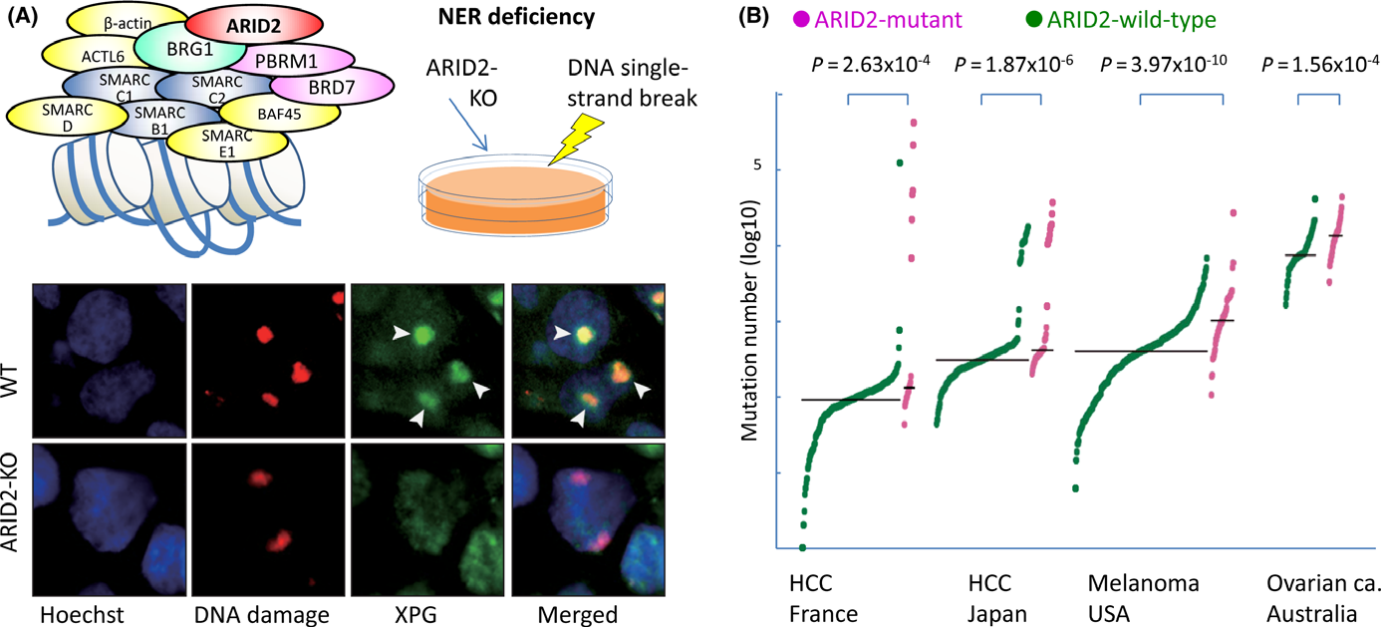
Disruption of DNA damage response and hypermutation in *ARID2*‐mutated hepatocellular carcinoma (HCC).^50^ A, Disruption of the recruitment of XPG to DNA‐damaged sites as a result of knock‐out of ARID2, a component of the SWI/SNF chromatin remodeling complex. B, Frequencies of genetic mutations in tumor samples from public data provided by the International Cancer Genome Consortium

Although frequent inactivating mutations were detected in an aggressive subtype of HCC (Figure 4B), it is still not understood how ARID2 plays tumor suppressor roles in cancer evolution. Recently, we used CRISPR/Cas9 genome‐editing technology to establish human HCC cells knocked out for the *ARID2* gene.^50^ ARID2 depletion attenuated nucleotide excision repair (NER) of DNA damage sites introduced by exposure to ultraviolet (UV) light as well as to chemical carcinogens, as XPG could not be accumulated without ARID2 (Figure 6A). By using large‐scale public data sets, we validated that ARID2 knock‐out could lead to similar molecular changes in vivo and, moreover, observed a higher number of somatic mutations in ARID2‐mutated subtypes than in the ARID2 wild‐type across various types of cancers including HCC (Figure 6B).

Our CRISPR‐mediated knock‐out for the *ARID2* gene provided evidence that the NER process is disrupted through inhibition of the recruitment of XPG, resulting in susceptibility to carcinogens and potential hypermutation in the *ARID2*‐mutated subtype of HCC. These findings present far‐reaching implications for therapeutic targets in cancers harboring *ARID2* mutations.^51^ The development of cancer immunotherapy has reached an important inflection point in the history of cancer therapy,^52^ and the correlation of a higher mutational load and a higher rate of response to immune checkpoint inhibitors has been shown.^53^ Such novel conceptual drugs may also be good candidates for ARID2‐mutated cancers displaying a hypermutator phenotype.

**Figure 7:**
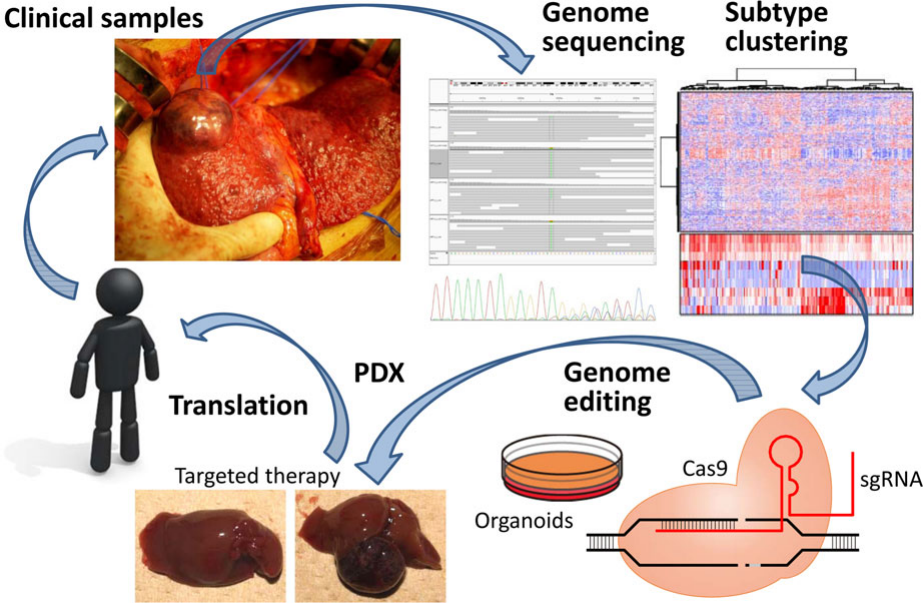
Precision medicine based on surgical oncology. Surgical tissues provide potential reserves of genomics, epigenomics, transcriptomics, metabolomics, proteomics and phenomic information as well as resources for organoids and patient‐derived xenografts (PDX). After genome‐integrated analysis of clinical samples, subtype clustering can be identified. Then, there are two ways of subtype‐specific treatment translated from (1) the genome editing/engineering models and (2) PDX or organoids for targeted therapy. Additionally, the clustered regularly interspaced short palindromic repeats (CRISPR)/Cas9 system can increase the effect of cancer immunotherapy. Genome editing technology can revolutionize precision cancer medicine in the translational circuit. sgRNA, short single‐guide RNA. Fee illustration derived from https://pictogram-free.com/

In addition to next‐generation genomic analysis, integrated epigenomic and transcriptomic analyses identified that several molecular subtypes exist in human HCC,^54^ pancreatic cancer,^55^ biliary cancer,^56^ and gastric cancer^57^ as shown in Table 1. Bulk tumor tissues are useful for clustering to better understand the transcriptional networks and molecular mechanisms that underpin the tumor microenvironment. In HCC, the Aurora mitotic pathway with *TP53* mutations characterizes the specific subtype that might be similar to the PLK1‐rich cluster reported by the TIGER‐LC Consortium.^58^ This subtype of HCC with mitotic instability shows poor prognosis, and contains a CSC‐rich and potentially immunogenic group.^59^, ^60^ In contrast, a less mitotic subtype of HCC is characterized by GLUL/FABP biomarkers, and composed of a *CTNNB1*‐mutated group with hypermethylation and an obesity group with immunogenic potential.^60^ In other cancer subtypes, poor prognosis is observed in *TP53/KDM6A*‐mutated pancreatic cancer and immunogenic biliary cancer with hypermutation (Table 1). Diffuse‐subtype of scirrhous gastric cancer is characterized by *CDH1*/*RHOA* mutations. In our laboratory, a unique genetically engineered mouse model of scirrhous gastric cancer was established by using double conditional knock‐out of *CDH1* and *Trp53* genes.^61^ Discovery of anticancer agents targeting cancer cells with genetic mutations is strategized by exploitation of the structure‐ability relationship and synthetic lethality.^62^, ^63^ Patient‐derived xenograft (PDX) and organoid models also provide potentially valuable information for estimating patient response to a given treatment, but there are some limitations to determine immunotherapy including checkpoint inhibitors.^64^ Genome‐scale analysis allows the identification of subtype clustering, and subtype‐specific treatment could then be translated not only from PDX/organoids, but also from genome editing/engineering models for targeted therapy (Figure 7). Precise characterization of the molecular subtypes encompassing tumor, stromal and immune components should uncover multi‐molecular additions that promise future perspectives for the development of precision cancer medicine.

**Table 1:** Molecular subtype classification of human cancers

HCC	Mitotic instability		Less mitotic	
	Aurora/PLKl		GLUL/FABP	
	TP53 mutation		CTNNB1 mutation	—
	EpCAM/CSC: Immune signal?	non‐CSC	Hypermethylation	Immune signal?
	Vascular invasion	—	—	Obesity
	Poor prognosis		Better prognosis	
Pancreatic cancer	Squamous/QM	ADEX	Progenitor	Immunogenic
	TP53/KDM6A mutation	—	TGFBR2 mutation	—
	TP63DNtargets	KRAS network	PDX1, HNF1/4	B cell signaling
	Poor prognosis	Exocrine	FOXA network	T cell signaling
Biliary cancer	Cluster 1	Cluster 2	Cluster 3	Cluster 4
	TP53/KRAS/SMAD4 mut.	TP53/KRAS/SMAD4 mut.	BAP1/IDH1/NRAS mut.	TP53/KRAS/SMAD4 mut.
	Extrahepatic	—	Intrahepatic	Hypermutation
	RAS/MAPK signal	—	FGFR2 fusion	Immune signal
	Better prognosis	—	—	Poor prognosis
Gastric cancer	EBV	MSI	GS	CIN
	PIK3CA mutation	Miscellaneous	CDH1/RHOA mutation	TP53 mutation
	CDKN2A silencing	MLH1 silencing	—	—
	Immune signal	RAS/PI3K signal	Cell adhesion pathways	RTK‐RAS signal
	CIMP	CIMP‐hypermutation	Diffuse type	Intestinal type

ADEX, aberrantly differentiated endocrine exocrine; CIMP, CpG island methylator phenotype; CIN, chromosomal instability; EBV, Epstein‐Barr virus; GS, genomic stability; HCC, hepatocellular carcinoma; MSI, microsatellite instability; QM, quasi‐mesenchymal; —, not significant.

## 5 CONCLUDING REMARKS

Emerging innovations of genome editing technology have extended to chromosomal rearrangements using two different sgRNAs guiding Cas9 to induce DNA cleavage at two different genes, large chromosomal deletions using two proximate sgRNAs guiding Cas9 to induce DNA cleavage at two different loci of the same gene,^65^ transcriptional control and even epigenetic modulation of specific genetic loci using nuclease‐inactivated version of Cas9 (dead‐Cas9; dCas9) that can be fused to different functional enzymatic domains such as translational regulator and epigenetic modifier, respectively.^66^ As proof of principle studies, multiplex CRISPR‐mediated genomic, epigenomic and transcriptomic modifications can be carried out to model functional consequences of molecular subtypes, to inhibit cancer by inactivating driver mutations, and to discover cancer drug targets by synthetic lethal interactions.^67^ In addition, genome editing can increase the effect of cancer immunotherapy.^68^ Further revolution of the CRISPR/Cas9 system can innovate precision cancer medicine in the near future.^69^

## DISCLOSURE

Conflicts of Interest: Author declares no conflicts of interest for this article.
